# Tetra­kis[bis­(pyridin-2-yl)amine-κ*N* ^2^](nitrato-κ*O*)silver(I)

**DOI:** 10.1107/S1600536814000907

**Published:** 2014-01-22

**Authors:** Yuliia Parashchenko, Anna Pavlishchuk, Nadezhda A. Bokach, Matti Haukka

**Affiliations:** a Department of Chemistry, Kiev National Taras Shevchenko University, Volodymyrska Street 62, Kiev 01601, Ukraine; b Department of Chemistry, Saint Petersburg State University, Universitetsky Pr. 26, 198504 Stary Petergof, Russian Federation; c Department of Chemistry, University of Joensuu, PO Box 111, FI-80108 Joensuu, Finland

## Abstract

In the title complex, [Ag(NO_3_)(C_10_H_9_N_3_)_4_], the nitrate ligand is found to be disordered over two sets of positions, with occupancy factors of 0.473 (5) and 0.527 (5). The Ag^I^ ion is located in a square-pyramidal coordination environment formed by four N atoms from four bis­(pyridin-2-yl)amine ligands and one O atom from a nitrate ligand. Weak inter­actions between the Ag^I^ ions and the nitrate anions acting in a monodentate mode [Ag⋯O = 2.791 (13) and 2.816 (9) Å for the major component of the nitrate ligand, and 2.865 (8) and 2.837 (8) Å for the minor component] link the complex mol­ecules into a chain along [001]. N—H⋯O hydrogen bonds are observed.

## Related literature   

For the use of silver complexes in medicine, see: Kascatan-Nebioglu *et al.* (2007 ▶); Kasuga *et al.* (2006 ▶). For the use of silver complexes as functional materials, see: Park *et al.* (2011 ▶); Takeuchi *et al.* (2001 ▶). For the ligand synthesis, see: Wibaut & Dingemanse (1923 ▶). For related structures, see: Fritsky *et al.* (2006 ▶); Jing *et al.* (2011 ▶); Moroz *et al.* (2012 ▶); Penkova *et al.* (2009 ▶); Zhang & Yang (2011 ▶).
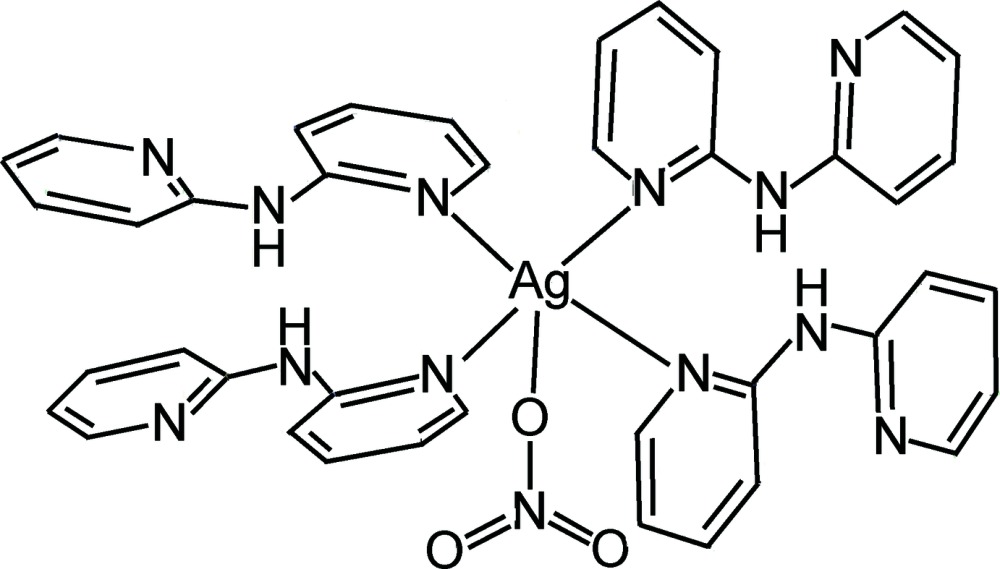


## Experimental   

### 

#### Crystal data  

[Ag(NO_3_)(C_10_H_9_N_3_)_4_]
*M* *_r_* = 854.69
Monoclinic, 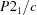

*a* = 12.2801 (16) Å
*b* = 23.038 (3) Å
*c* = 13.7091 (16) Å
β = 104.499 (4)°
*V* = 3754.9 (8) Å^3^
*Z* = 4
Mo *K*α radiation
μ = 0.60 mm^−1^
*T* = 100 K
0.29 × 0.06 × 0.03 mm

#### Data collection  

Bruker APEXII CCD diffractometer
Absorption correction: multi-scan (*SADABS*; Sheldrick, 1996 ▶) *T*_min_ = 0.958, *T*_max_ = 0.982
11456 measured reflections
6643 independent reflections
3690 reflections with *I* > 2σ(*I*)
*R*_int_ = 0.049

#### Refinement  

*R*[*F* ^2^ > 2σ(*F* ^2^)] = 0.061
*wR*(*F* ^2^) = 0.151
*S* = 1.01
6643 reflections
509 parameters
29 restraints
H-atom parameters constrained
Δρ_max_ = 1.05 e Å^−3^
Δρ_min_ = −2.19 e Å^−3^

### 

Data collection: *APEX2* (Bruker, 2007 ▶); cell refinement: *SAINT* (Bruker, 2007 ▶); data reduction: *SAINT*; program(s) used to solve structure: *SIR2004* (Burla *et al.*, 2005 ▶); program(s) used to refine structure: *SHELXL97* (Sheldrick, 2008 ▶); molecular graphics: *Mercury* (Macrae *et al.*, 2008 ▶); software used to prepare material for publication: *publCIF* (Westrip, 2010 ▶).

## Supplementary Material

Crystal structure: contains datablock(s) I. DOI: 10.1107/S1600536814000907/hy2640sup1.cif

Structure factors: contains datablock(s) I. DOI: 10.1107/S1600536814000907/hy2640Isup2.hkl

CCDC reference:

Additional supporting information:  crystallographic information; 3D view; checkCIF report

## Figures and Tables

**Table 1:** Hydrogen-bond geometry (Å, °)

*D*—H⋯*A*	*D*—H	H⋯*A*	*D*⋯*A*	*D*—H⋯*A*
N2—H2⋯O1*B* ^i^	0.88	2.15	2.959 (14)	152
N2—H2⋯O1*A* ^i^	0.88	2.27	3.089 (16)	155
N5—H5⋯O3*B*	0.88	2.22	3.075 (10)	163
N5—H5⋯O2*A*	0.88	2.40	3.271 (11)	172
N8—H8⋯O3*A* ^i^	0.88	2.21	3.073 (12)	168
N11—H11⋯O2*B*	0.88	2.26	3.129 (10)	168
N11—H11⋯O2*A*	0.88	2.27	3.093 (11)	156

Symmetry code: (i) 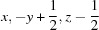.

**Fig. 1.**
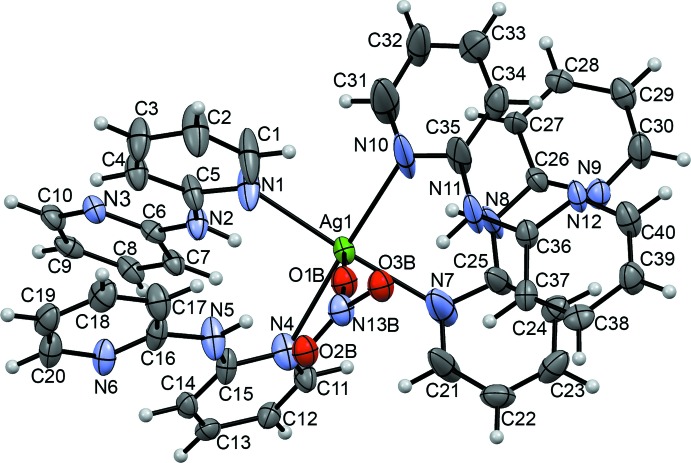
The molecular structure of the title complex. Displacement ellipsoids are drawn at the 50% probability level. The minor disordered fraction has been omitted for clarity.

**Fig. 2.**
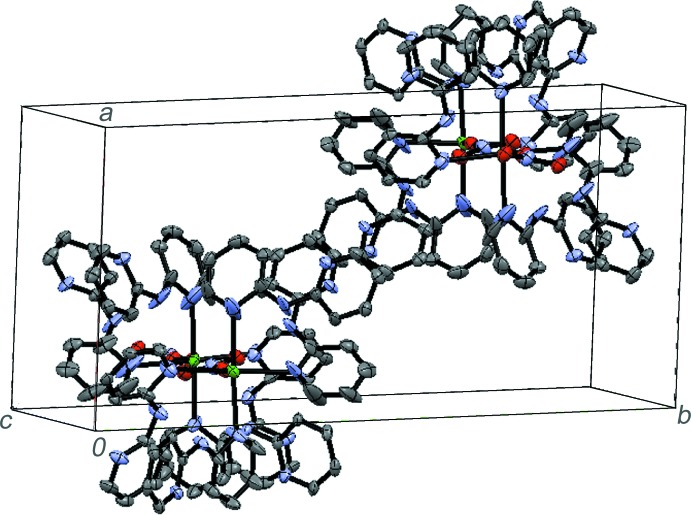
The unit cell of the title complex. H atoms and minor disordered atoms has been omitted for clarity.

## supplementary crystallographic information

### 1. Comment

Some of silver compounds are proved to be useful in medicine. The silver complexes display antimicrobial activities against bacteria, yeasts and molds (Kascatan-Nebioglu *et al.*, 2007; Kasuga *et al.*, 2006). Silver complexes also display conductivity, luminescence and photoluminescence (Park *et al.*, 2011; Takeuchi *et al.*, 2001). In this study we have chosen bis(pyridin-2-yl)amine (dipam) as a ligand.

The preparation of dipam was reported by Wibaut & Dingemanse (1923) and since that time it was widely used for constructing complexes with transition metals. Two crystalline modifications of the compound C_10_H_9_N_3_ are known, one with melting point at 84°, while a second melts at 94°C. In this paper we report the synthesis and characterization of the title compound. The nitrate anion in the complex is disordered between two sets of positions [occupancy factors are equal to 0.473 (5) and 0.527 (5)]. In both cases coordination environment of the Ag^I^ ion is formed by four N atoms from four dipam ligands [Ag—N distances fall in a range of 2.420 (9)–2.532 (9) Å]. On the contrary to the already reported compounds, the title complex contains monodentately coordinated dipam ligands. The coordinated environment of the pentacoordinated Ag^I^ ion is completed by an O atom (O2A or O1B) from the nitrate anion [Ag1—O2A = 2.511 (8), Ag1—O1B^i^ = 2.648 (12) Å. Symmetry code: (i) x, 1/2-y, -1/2+z]. The observed metal–ligand bond distances are typical for silver(I) complexes (Jing *et al.*, 2011; Zhang & Yang, 2011). The Ag^I^ ion in the complex slightly deviates (0.061 Å) from the mean plane formed by the coordinated N atoms (N1, N4, N7, N10) from four dipam ligands. The C—N and C—C bond lengths in the pyridine rings are normal for 2-substituted pyridine derivatives (Fritsky *et al.*, 2006; Moroz *et al.*, 2012; Penkova *et al.*, 2009).

### 2. Experimental

A solution of silver(I) nitrate (42 mg, 0.25 mmol) in methanol (5 ml) was added to a solution of bis(pyridin-2-yl)amine (171 mg, 1 mmol) in methanol (10 ml). Resulting mixture was stirred for 1 h. After filtering, the filtrate was left for a slow evaporation. Colorless crystals of the title compound, which formed during one week, were filtered out and air dried (yield: 152 mg, 21%). Analysis, calculated for C_40_H_36_AgN_13_O_3_: C 56.2, H 4.2, N 21.3%; found: C 56.6, H 3.8, N 21.6%.

### 3. Refinement

The nitrate anion was disordered over two sets of sites with occupancies 0.527 (5) and 0.473 (5). The N—O and O—O distances as well as the anisotropic displacement parameters of the N and O atoms within these disordered anions were restrained to be similar. Furthermore, the geometry of the nitrate anion was restrained to be planar. One of the pyridyl N atoms (N7) was restrained so that its *U*_ij_ components approximate to isotropic behavior. H atoms were positioned geometrically and refined as riding atoms, with C—H = 0.95 and N—H = 0.88 Å and with *U*_iso_(H) = 1.2*U*_eq_(C, N). The highest residual peak is located 0.30 Å from atom O3A and the deepest hole is located 0.12 Å from atom O2A.

### Crystal data

**Table:**

[Ag(NO_3_)(C_10_H_9_N_3_)_4_]	*F*(000) = 1752
*M**_r_* = 854.69	*D*_x_ = 1.512 Mg m^−^^3^
Monoclinic, *P*2_1_/*c*	Mo *K*α radiation, λ = 0.71073 Å
Hall symbol: -P 2ybc	Cell parameters from 1361 reflections
*a* = 12.2801 (16) Å	θ = 2.3–21.1°
*b* = 23.038 (3) Å	µ = 0.60 mm^−^^1^
*c* = 13.7091 (16) Å	*T* = 100 K
β = 104.499 (4)°	Needle, colourless
*V* = 3754.9 (8) Å^3^	0.29 × 0.06 × 0.03 mm
*Z* = 4	

### Data collection

**Table:**

Bruker APEXII CCD diffractometer	6643 independent reflections
Radiation source: fine-focus sealed tube	3690 reflections with *I* > 2σ(*I*)
Flat graphite crystal monochromator	*R*_int_ = 0.049
Detector resolution: 16 pixels mm^-1^	θ_max_ = 25.1°, θ_min_ = 1.8°
φ and ω scans	*h* = −11→14
Absorption correction: multi-scan (*SADABS*; Sheldrick, 1996)	*k* = −15→27
*T*_min_ = 0.958, *T*_max_ = 0.982	*l* = −16→13
11456 measured reflections	

### Refinement

**Table:**

Refinement on *F*^2^	Primary atom site location: structure-invariant direct methods
Least-squares matrix: full	Secondary atom site location: difference Fourier map
*R*[*F*^2^ > 2σ(*F*^2^)] = 0.061	Hydrogen site location: inferred from neighbouring sites
*wR*(*F*^2^) = 0.151	H-atom parameters constrained
*S* = 1.01	*w* = 1/[σ^2^(*F*_o_^2^) + (0.0609*P*)^2^ + 2.4207*P*] where *P* = (*F*_o_^2^ + 2*F*_c_^2^)/3
6643 reflections	(Δ/σ)_max_ < 0.001
509 parameters	Δρ_max_ = 1.05 e Å^−^^3^
29 restraints	Δρ_min_ = −2.19 e Å^−^^3^

### Special details

**Table:**

Geometry. All e.s.d.'s (except the e.s.d. in the dihedral angle between two l.s. planes) are estimated using the full covariance matrix. The cell e.s.d.'s are taken into account individually in the estimation of e.s.d.'s in distances, angles and torsion angles; correlations between e.s.d.'s in cell parameters are only used when they are defined by crystal symmetry. An approximate (isotropic) treatment of cell e.s.d.'s is used for estimating e.s.d.'s involving l.s. planes.
Refinement. Refinement of *F*^2^ against ALL reflections. The weighted *R*-factor *wR* and goodness of fit *S* are based on *F*^2^, conventional *R*-factors *R* are based on *F*, with *F* set to zero for negative *F*^2^. The threshold expression of *F*^2^ > σ(*F*^2^) is used only for calculating *R*-factors(gt) *etc*. and is not relevant to the choice of reflections for refinement. *R*-factors based on *F*^2^ are statistically about twice as large as those based on *F*, and *R*- factors based on ALL data will be even larger.

### Fractional atomic coordinates and isotropic or equivalent isotropic displacement parameters (Å^2^)

**Table:**

	*x*	*y*	*z*	*U*_iso_*/*U*_eq_	Occ. (<1)
Ag1	0.17127 (4)	0.24786 (2)	0.08079 (4)	0.02838 (16)	
N13A	0.1669 (9)	0.2518 (5)	0.3523 (7)	0.0369 (9)	0.473 (5)
O1A	0.1909 (13)	0.2997 (5)	0.3987 (10)	0.0369 (9)	0.473 (5)
O2A	0.1631 (9)	0.2582 (4)	0.2612 (6)	0.0369 (9)	0.473 (5)
O3A	0.1481 (8)	0.2072 (4)	0.3856 (7)	0.0369 (9)	0.473 (5)
N13B	0.1685 (9)	0.2611 (4)	0.3255 (7)	0.0369 (9)	0.527 (5)
O1B	0.1745 (11)	0.2885 (5)	0.3983 (9)	0.0369 (9)	0.527 (5)
O2B	0.2496 (7)	0.2419 (4)	0.2957 (6)	0.0369 (9)	0.527 (5)
O3B	0.0812 (7)	0.2619 (4)	0.2508 (6)	0.0369 (9)	0.527 (5)
N1	−0.0337 (5)	0.2501 (3)	0.0273 (5)	0.0564 (19)	
N2	−0.0488 (5)	0.1740 (2)	−0.0804 (4)	0.0340 (14)	
H2	0.0246	0.1784	−0.0666	0.041*	
N3	−0.2006 (5)	0.1208 (2)	−0.1780 (4)	0.0303 (14)	
N4	0.1850 (5)	0.1399 (3)	0.1059 (5)	0.0432 (17)	
N5	0.0397 (5)	0.1353 (3)	0.1806 (5)	0.0455 (17)	
H5	0.0669	0.1693	0.2037	0.055*	
N6	−0.1073 (5)	0.0700 (3)	0.1722 (4)	0.0376 (15)	
N7	0.3748 (7)	0.2493 (3)	0.1219 (6)	0.093 (3)	
N8	0.3872 (6)	0.3217 (3)	0.0092 (6)	0.085 (3)	
H8	0.3194	0.3089	−0.0202	0.102*	
N9	0.5243 (5)	0.3905 (3)	−0.0021 (5)	0.0366 (15)	
N10	0.1604 (7)	0.3575 (4)	0.0857 (6)	0.097 (4)	
N11	0.2960 (6)	0.3680 (3)	0.2315 (5)	0.061 (2)	
H11	0.2777	0.3318	0.2403	0.073*	
N12	0.4227 (5)	0.4422 (2)	0.2989 (4)	0.0365 (15)	
C1	−0.0789 (7)	0.2885 (4)	0.0804 (8)	0.075 (3)	
H1	−0.0312	0.3175	0.1179	0.090*	
C2	−0.1912 (7)	0.2880 (4)	0.0834 (8)	0.069 (3)	
H2A	−0.2207	0.3164	0.1200	0.082*	
C3	−0.2571 (6)	0.2447 (3)	0.0315 (6)	0.052 (2)	
H3	−0.3340	0.2425	0.0324	0.062*	
C4	−0.2129 (6)	0.2042 (3)	−0.0227 (6)	0.0403 (19)	
H4	−0.2579	0.1732	−0.0568	0.048*	
C5	−0.1022 (6)	0.2099 (3)	−0.0257 (6)	0.0362 (19)	
C6	−0.0910 (6)	0.1323 (3)	−0.1533 (5)	0.0268 (15)	
C7	−0.0146 (6)	0.1047 (3)	−0.1987 (5)	0.0268 (15)	
H7	0.0634	0.1136	−0.1790	0.032*	
C8	−0.0560 (6)	0.0645 (3)	−0.2724 (5)	0.0318 (17)	
H8A	−0.0067	0.0453	−0.3053	0.038*	
C9	−0.1701 (6)	0.0521 (3)	−0.2987 (5)	0.0305 (17)	
H9	−0.2000	0.0242	−0.3493	0.037*	
C10	−0.2385 (6)	0.0807 (3)	−0.2507 (5)	0.0332 (18)	
H10	−0.3167	0.0722	−0.2691	0.040*	
C11	0.2549 (6)	0.1156 (3)	0.0550 (6)	0.0358 (18)	
H11A	0.3122	0.1393	0.0403	0.043*	
C12	0.2479 (5)	0.0602 (3)	0.0239 (5)	0.0306 (16)	
H12	0.2995	0.0450	−0.0108	0.037*	
C13	0.1633 (6)	0.0254 (3)	0.0438 (5)	0.0324 (18)	
H13	0.1549	−0.0137	0.0212	0.039*	
C14	0.0918 (5)	0.0486 (3)	0.0969 (5)	0.0298 (16)	
H14	0.0348	0.0253	0.1129	0.036*	
C15	0.1037 (6)	0.1056 (3)	0.1262 (5)	0.0349 (18)	
C16	−0.0589 (6)	0.1202 (3)	0.2041 (5)	0.0355 (18)	
C17	−0.1048 (6)	0.1600 (3)	0.2615 (6)	0.043 (2)	
H17	−0.0673	0.1955	0.2834	0.051*	
C18	−0.2037 (6)	0.1468 (3)	0.2850 (6)	0.044 (2)	
H18	−0.2362	0.1725	0.3240	0.053*	
C19	−0.2555 (6)	0.0941 (3)	0.2499 (6)	0.042 (2)	
H19	−0.3252	0.0837	0.2632	0.050*	
C20	−0.2045 (6)	0.0583 (4)	0.1966 (6)	0.044 (2)	
H20	−0.2400	0.0223	0.1748	0.053*	
C21	0.4230 (9)	0.2173 (4)	0.2021 (7)	0.083 (4)	
H21	0.3813	0.1852	0.2171	0.100*	
C22	0.5258 (7)	0.2263 (3)	0.2637 (6)	0.047 (2)	
H22	0.5548	0.2025	0.3209	0.056*	
C23	0.5858 (6)	0.2714 (4)	0.2398 (6)	0.049 (2)	
H23	0.6575	0.2800	0.2827	0.059*	
C24	0.5457 (6)	0.3047 (3)	0.1557 (5)	0.0355 (17)	
H24	0.5890	0.3356	0.1390	0.043*	
C25	0.4404 (7)	0.2919 (3)	0.0961 (6)	0.051 (2)	
C26	0.4229 (6)	0.3683 (3)	−0.0391 (6)	0.0351 (18)	
C27	0.3477 (6)	0.3905 (3)	−0.1247 (6)	0.042 (2)	
H27	0.2746	0.3743	−0.1484	0.050*	
C28	0.3816 (6)	0.4356 (3)	−0.1732 (5)	0.0313 (17)	
H28	0.3333	0.4504	−0.2333	0.038*	
C29	0.4856 (6)	0.4602 (3)	−0.1358 (6)	0.039 (2)	
H29	0.5097	0.4927	−0.1675	0.047*	
C30	0.5527 (7)	0.4360 (4)	−0.0511 (6)	0.049 (2)	
H30	0.6249	0.4526	−0.0250	0.059*	
C31	0.0905 (9)	0.3789 (5)	0.0006 (8)	0.113 (5)	
H31	0.0403	0.3528	−0.0421	0.136*	
C32	0.0884 (8)	0.4364 (5)	−0.0274 (7)	0.085 (4)	
H32	0.0382	0.4503	−0.0873	0.102*	
C33	0.1623 (7)	0.4723 (4)	0.0356 (7)	0.053 (2)	
H33	0.1632	0.5124	0.0194	0.064*	
C34	0.2357 (6)	0.4522 (4)	0.1221 (6)	0.044 (2)	
H34	0.2877	0.4774	0.1648	0.053*	
C35	0.2309 (7)	0.3937 (4)	0.1445 (6)	0.060 (3)	
C36	0.3838 (6)	0.3890 (3)	0.3064 (5)	0.0314 (16)	
C37	0.4302 (5)	0.3523 (3)	0.3872 (5)	0.0323 (17)	
H37	0.4012	0.3143	0.3898	0.039*	
C38	0.5182 (6)	0.3718 (3)	0.4631 (6)	0.0348 (18)	
H38	0.5500	0.3480	0.5196	0.042*	
C39	0.5595 (7)	0.4273 (3)	0.4548 (6)	0.043 (2)	
H39	0.6208	0.4423	0.5052	0.051*	
C40	0.5100 (7)	0.4597 (3)	0.3725 (6)	0.043 (2)	
H40	0.5395	0.4974	0.3670	0.051*	

### Atomic displacement parameters (Å^2^)

**Table:**

	*U*^11^	*U*^22^	*U*^33^	*U*^12^	*U*^13^	*U*^23^
Ag1	0.0302 (3)	0.0252 (3)	0.0316 (3)	−0.0075 (3)	0.01118 (19)	−0.0037 (3)
N13A	0.0408 (19)	0.038 (2)	0.0324 (19)	−0.008 (2)	0.0101 (18)	−0.0068 (19)
O1A	0.0408 (19)	0.038 (2)	0.0324 (19)	−0.008 (2)	0.0101 (18)	−0.0068 (19)
O2A	0.0408 (19)	0.038 (2)	0.0324 (19)	−0.008 (2)	0.0101 (18)	−0.0068 (19)
O3A	0.0408 (19)	0.038 (2)	0.0324 (19)	−0.008 (2)	0.0101 (18)	−0.0068 (19)
N13B	0.0408 (19)	0.038 (2)	0.0324 (19)	−0.008 (2)	0.0101 (18)	−0.0068 (19)
O1B	0.0408 (19)	0.038 (2)	0.0324 (19)	−0.008 (2)	0.0101 (18)	−0.0068 (19)
O2B	0.0408 (19)	0.038 (2)	0.0324 (19)	−0.008 (2)	0.0101 (18)	−0.0068 (19)
O3B	0.0408 (19)	0.038 (2)	0.0324 (19)	−0.008 (2)	0.0101 (18)	−0.0068 (19)
N1	0.040 (3)	0.052 (4)	0.091 (5)	−0.021 (4)	0.043 (4)	−0.044 (5)
N2	0.029 (3)	0.032 (3)	0.045 (4)	−0.009 (3)	0.018 (3)	−0.017 (3)
N3	0.032 (3)	0.026 (3)	0.034 (4)	−0.007 (3)	0.012 (3)	−0.002 (3)
N4	0.048 (4)	0.043 (4)	0.044 (4)	−0.011 (3)	0.023 (4)	−0.014 (3)
N5	0.046 (4)	0.051 (4)	0.049 (4)	−0.023 (3)	0.031 (3)	−0.028 (3)
N6	0.034 (4)	0.054 (4)	0.027 (3)	−0.015 (3)	0.013 (3)	−0.008 (3)
N7	0.100 (5)	0.065 (4)	0.077 (5)	−0.048 (5)	−0.045 (4)	0.034 (5)
N8	0.080 (6)	0.055 (4)	0.082 (6)	−0.053 (4)	−0.052 (4)	0.047 (4)
N9	0.031 (3)	0.043 (4)	0.036 (4)	−0.009 (3)	0.008 (3)	0.004 (3)
N10	0.094 (7)	0.099 (6)	0.068 (6)	−0.080 (6)	−0.038 (5)	0.050 (5)
N11	0.067 (5)	0.064 (5)	0.038 (4)	−0.051 (4)	−0.013 (4)	0.019 (4)
N12	0.037 (4)	0.036 (3)	0.037 (4)	−0.020 (3)	0.009 (3)	0.001 (3)
C1	0.057 (6)	0.065 (6)	0.120 (9)	−0.041 (5)	0.056 (6)	−0.064 (6)
C2	0.059 (6)	0.064 (6)	0.100 (8)	−0.029 (5)	0.051 (6)	−0.053 (6)
C3	0.035 (4)	0.058 (5)	0.071 (5)	−0.017 (4)	0.030 (4)	−0.031 (5)
C4	0.040 (4)	0.038 (4)	0.049 (5)	−0.014 (3)	0.023 (4)	−0.019 (4)
C5	0.038 (4)	0.029 (4)	0.051 (5)	−0.015 (3)	0.029 (4)	−0.018 (4)
C6	0.036 (4)	0.018 (3)	0.029 (4)	0.000 (3)	0.013 (3)	−0.001 (3)
C7	0.031 (4)	0.019 (3)	0.033 (4)	−0.003 (3)	0.012 (3)	−0.006 (3)
C8	0.044 (5)	0.023 (4)	0.034 (4)	−0.004 (3)	0.021 (4)	−0.002 (3)
C9	0.051 (5)	0.019 (3)	0.021 (4)	0.000 (3)	0.007 (4)	−0.001 (3)
C10	0.041 (5)	0.019 (3)	0.038 (5)	−0.005 (3)	0.007 (4)	−0.004 (3)
C11	0.029 (4)	0.035 (4)	0.048 (5)	−0.006 (3)	0.019 (4)	−0.002 (4)
C12	0.024 (4)	0.034 (4)	0.034 (4)	−0.002 (3)	0.005 (3)	−0.004 (3)
C13	0.030 (4)	0.029 (4)	0.031 (4)	0.002 (3)	−0.004 (4)	0.004 (3)
C14	0.026 (4)	0.035 (4)	0.029 (4)	−0.007 (3)	0.008 (3)	0.007 (3)
C15	0.028 (4)	0.051 (5)	0.027 (4)	−0.017 (4)	0.011 (3)	−0.011 (4)
C16	0.025 (4)	0.057 (5)	0.025 (4)	−0.013 (3)	0.008 (3)	−0.002 (4)
C17	0.045 (5)	0.047 (5)	0.042 (5)	−0.015 (4)	0.021 (4)	−0.010 (4)
C18	0.036 (4)	0.059 (5)	0.042 (5)	−0.002 (4)	0.019 (4)	0.004 (4)
C19	0.028 (4)	0.062 (6)	0.037 (5)	−0.006 (4)	0.012 (4)	0.012 (4)
C20	0.032 (4)	0.061 (5)	0.037 (5)	−0.018 (4)	0.005 (4)	−0.003 (4)
C21	0.109 (9)	0.046 (5)	0.057 (6)	−0.044 (5)	−0.049 (6)	0.024 (5)
C22	0.054 (5)	0.038 (4)	0.042 (5)	0.010 (4)	0.002 (4)	0.012 (4)
C23	0.030 (4)	0.076 (6)	0.035 (5)	0.004 (4)	−0.001 (4)	0.006 (4)
C24	0.029 (4)	0.043 (4)	0.035 (4)	0.002 (3)	0.009 (3)	0.002 (4)
C25	0.063 (6)	0.031 (4)	0.041 (5)	−0.019 (4)	−0.023 (4)	0.009 (4)
C26	0.038 (4)	0.021 (4)	0.041 (5)	−0.008 (3)	0.000 (4)	0.003 (3)
C27	0.033 (4)	0.033 (4)	0.050 (5)	−0.009 (3)	−0.010 (4)	0.020 (4)
C28	0.036 (4)	0.029 (4)	0.030 (4)	0.002 (3)	0.009 (3)	0.004 (3)
C29	0.046 (5)	0.038 (4)	0.038 (5)	−0.011 (4)	0.018 (4)	0.003 (4)
C30	0.040 (5)	0.061 (6)	0.044 (6)	−0.024 (4)	0.005 (4)	0.006 (5)
C31	0.101 (9)	0.120 (10)	0.080 (8)	−0.073 (8)	−0.052 (7)	0.058 (7)
C32	0.055 (6)	0.117 (9)	0.065 (7)	−0.050 (6)	−0.017 (5)	0.053 (6)
C33	0.040 (5)	0.075 (6)	0.050 (6)	−0.003 (5)	0.021 (5)	0.031 (5)
C34	0.034 (4)	0.061 (5)	0.040 (5)	−0.014 (4)	0.011 (4)	0.009 (4)
C35	0.057 (6)	0.077 (6)	0.039 (5)	−0.036 (5)	0.001 (4)	0.034 (5)
C36	0.032 (4)	0.035 (4)	0.027 (4)	−0.010 (3)	0.007 (3)	0.000 (3)
C37	0.029 (4)	0.036 (4)	0.033 (4)	−0.009 (3)	0.010 (3)	−0.007 (3)
C38	0.036 (4)	0.037 (4)	0.033 (4)	0.009 (3)	0.013 (4)	−0.003 (3)
C39	0.044 (5)	0.043 (5)	0.036 (5)	−0.015 (4)	0.002 (4)	−0.003 (4)
C40	0.054 (5)	0.043 (5)	0.032 (5)	−0.021 (4)	0.013 (4)	−0.003 (4)

### Geometric parameters (Å, º)

**Table:**

Ag1—N7	2.420 (9)	C8—H8A	0.9500
Ag1—N1	2.439 (6)	C9—C10	1.361 (9)
Ag1—N4	2.511 (6)	C9—H9	0.9500
Ag1—N10	2.532 (9)	C10—H10	0.9500
Ag1—O2A	2.511 (8)	C11—C12	1.343 (9)
Ag1—O1B^i^	2.648 (12)	C11—H11A	0.9500
N13A—O3A	1.172 (11)	C12—C13	1.392 (9)
N13A—O2A	1.247 (11)	C12—H12	0.9500
N13A—O1A	1.269 (12)	C13—C14	1.381 (9)
N13B—O1B	1.168 (11)	C13—H13	0.9500
N13B—O2B	1.248 (10)	C14—C15	1.372 (9)
N13B—O3B	1.284 (11)	C14—H14	0.9500
N1—C5	1.337 (8)	C16—C17	1.414 (10)
N1—C1	1.350 (9)	C17—C18	1.367 (9)
N2—C5	1.386 (8)	C17—H17	0.9500
N2—C6	1.389 (8)	C18—C19	1.397 (10)
N2—H2	0.8800	C18—H18	0.9500
N3—C6	1.330 (8)	C19—C20	1.356 (10)
N3—C10	1.352 (8)	C19—H19	0.9500
N4—C11	1.355 (8)	C20—H20	0.9500
N4—C15	1.355 (8)	C21—C22	1.347 (11)
N5—C16	1.374 (8)	C21—H21	0.9500
N5—C15	1.391 (8)	C22—C23	1.360 (11)
N5—H5	0.8800	C22—H22	0.9500
N6—C16	1.325 (9)	C23—C24	1.371 (10)
N6—C20	1.344 (9)	C23—H23	0.9500
N7—C21	1.335 (10)	C24—C25	1.378 (10)
N7—C25	1.371 (10)	C24—H24	0.9500
N8—C25	1.388 (9)	C26—C27	1.395 (9)
N8—C26	1.390 (9)	C27—C28	1.355 (9)
N8—H8	0.8800	C27—H27	0.9500
N9—C26	1.325 (8)	C28—C29	1.373 (10)
N9—C30	1.337 (9)	C28—H28	0.9500
N10—C35	1.320 (10)	C29—C30	1.363 (11)
N10—C31	1.357 (11)	C29—H29	0.9500
N11—C36	1.377 (9)	C30—H30	0.9500
N11—C35	1.389 (10)	C31—C32	1.377 (13)
N11—H11	0.8800	C31—H31	0.9500
N12—C36	1.330 (8)	C32—C33	1.366 (12)
N12—C40	1.337 (9)	C32—H32	0.9500
C1—C2	1.390 (11)	C33—C34	1.378 (11)
C1—H1	0.9500	C33—H33	0.9500
C2—C3	1.366 (10)	C34—C35	1.387 (11)
C2—H2A	0.9500	C34—H34	0.9500
C3—C4	1.385 (9)	C36—C37	1.396 (9)
C3—H3	0.9500	C37—C38	1.374 (9)
C4—C5	1.377 (9)	C37—H37	0.9500
C4—H4	0.9500	C38—C39	1.391 (9)
C6—C7	1.401 (8)	C38—H38	0.9500
C7—C8	1.371 (9)	C39—C40	1.362 (10)
C7—H7	0.9500	C39—H39	0.9500
C8—C9	1.387 (9)	C40—H40	0.9500
			
N7—Ag1—N1	175.6 (3)	C12—C13—H13	120.5
N7—Ag1—N4	87.2 (2)	C15—C14—C13	119.3 (6)
N1—Ag1—N4	95.2 (2)	C15—C14—H14	120.3
N7—Ag1—O2A	93.6 (3)	C13—C14—H14	120.3
N1—Ag1—O2A	90.1 (3)	N4—C15—C14	121.9 (6)
N4—Ag1—O2A	88.7 (3)	N4—C15—N5	111.6 (6)
N7—Ag1—N10	92.2 (3)	C14—C15—N5	126.5 (6)
N1—Ag1—N10	86.0 (2)	N6—C16—N5	119.5 (6)
N4—Ag1—N10	170.8 (2)	N6—C16—C17	122.8 (6)
O2A—Ag1—N10	82.2 (3)	N5—C16—C17	117.7 (6)
O3A—N13A—O2A	122.1 (10)	C18—C17—C16	119.2 (7)
O3A—N13A—O1A	127.7 (10)	C18—C17—H17	120.4
O2A—N13A—O1A	110.2 (11)	C16—C17—H17	120.4
N13A—O2A—Ag1	167.1 (8)	C17—C18—C19	117.8 (7)
O1B—N13B—O2B	125.9 (11)	C17—C18—H18	121.1
O1B—N13B—O3B	122.3 (11)	C19—C18—H18	121.1
O2B—N13B—O3B	108.6 (8)	C20—C19—C18	118.9 (7)
C5—N1—C1	117.3 (6)	C20—C19—H19	120.6
C5—N1—Ag1	127.6 (5)	C18—C19—H19	120.6
C1—N1—Ag1	112.6 (5)	N6—C20—C19	124.8 (7)
C5—N2—C6	131.3 (6)	N6—C20—H20	117.6
C5—N2—H2	114.4	C19—C20—H20	117.6
C6—N2—H2	114.4	N7—C21—C22	125.7 (8)
C6—N3—C10	117.6 (6)	N7—C21—H21	117.1
C11—N4—C15	117.4 (6)	C22—C21—H21	117.1
C11—N4—Ag1	111.6 (4)	C21—C22—C23	116.5 (8)
C15—N4—Ag1	125.3 (5)	C21—C22—H22	121.8
C16—N5—C15	130.7 (6)	C23—C22—H22	121.8
C16—N5—H5	114.7	C22—C23—C24	121.9 (7)
C15—N5—H5	114.7	C22—C23—H23	119.0
C16—N6—C20	116.4 (6)	C24—C23—H23	119.0
C21—N7—C25	116.1 (7)	C23—C24—C25	117.7 (7)
C21—N7—Ag1	113.8 (7)	C23—C24—H24	121.2
C25—N7—Ag1	126.1 (6)	C25—C24—H24	121.2
C25—N8—C26	130.9 (7)	N7—C25—C24	121.7 (7)
C25—N8—H8	114.5	N7—C25—N8	113.3 (7)
C26—N8—H8	114.5	C24—C25—N8	124.9 (7)
C26—N9—C30	116.6 (7)	N9—C26—N8	119.6 (7)
C35—N10—C31	118.3 (9)	N9—C26—C27	122.8 (6)
C35—N10—Ag1	127.9 (7)	N8—C26—C27	117.6 (6)
C31—N10—Ag1	111.4 (7)	C28—C27—C26	118.2 (7)
C36—N11—C35	131.8 (7)	C28—C27—H27	120.9
C36—N11—H11	114.1	C26—C27—H27	120.9
C35—N11—H11	114.1	C27—C28—C29	120.3 (7)
C36—N12—C40	117.1 (6)	C27—C28—H28	119.8
N1—C1—C2	123.8 (7)	C29—C28—H28	119.8
N1—C1—H1	118.1	C30—C29—C28	117.1 (7)
C2—C1—H1	118.1	C30—C29—H29	121.4
C3—C2—C1	116.9 (7)	C28—C29—H29	121.4
C3—C2—H2A	121.5	N9—C30—C29	124.8 (7)
C1—C2—H2A	121.5	N9—C30—H30	117.6
C2—C3—C4	120.8 (7)	C29—C30—H30	117.6
C2—C3—H3	119.6	N10—C31—C32	123.5 (9)
C4—C3—H3	119.6	N10—C31—H31	118.3
C5—C4—C3	118.2 (6)	C32—C31—H31	118.3
C5—C4—H4	120.9	C33—C32—C31	116.4 (9)
C3—C4—H4	120.9	C33—C32—H32	121.8
N1—C5—C4	122.8 (6)	C31—C32—H32	121.8
N1—C5—N2	112.8 (6)	C32—C33—C34	121.9 (9)
C4—C5—N2	124.3 (6)	C32—C33—H33	119.1
N3—C6—N2	119.5 (6)	C34—C33—H33	119.1
N3—C6—C7	122.9 (6)	C33—C34—C35	117.6 (8)
N2—C6—C7	117.6 (6)	C33—C34—H34	121.2
C8—C7—C6	117.9 (6)	C35—C34—H34	121.2
C8—C7—H7	121.0	N10—C35—C34	122.3 (8)
C6—C7—H7	121.0	N10—C35—N11	113.9 (8)
C7—C8—C9	119.6 (6)	C34—C35—N11	123.8 (8)
C7—C8—H8A	120.2	N12—C36—N11	119.6 (6)
C9—C8—H8A	120.2	N12—C36—C37	122.4 (6)
C10—C9—C8	118.7 (6)	N11—C36—C37	117.9 (6)
C10—C9—H9	120.6	C38—C37—C36	119.4 (6)
C8—C9—H9	120.6	C38—C37—H37	120.3
N3—C10—C9	123.2 (7)	C36—C37—H37	120.3
N3—C10—H10	118.4	C37—C38—C39	118.2 (7)
C9—C10—H10	118.4	C37—C38—H38	120.9
C12—C11—N4	123.9 (6)	C39—C38—H38	120.9
C12—C11—H11A	118.1	C40—C39—C38	118.4 (7)
N4—C11—H11A	118.1	C40—C39—H39	120.8
C11—C12—C13	118.5 (6)	C38—C39—H39	120.8
C11—C12—H12	120.7	N12—C40—C39	124.6 (7)
C13—C12—H12	120.7	N12—C40—H40	117.7
C14—C13—C12	119.0 (6)	C39—C40—H40	117.7
C14—C13—H13	120.5		

Symmetry code: (i) *x*, −*y*+1/2, *z*−1/2.

### Hydrogen-bond geometry (Å, º)

**Table:**

*D*—H···*A*	*D*—H	H···*A*	*D*···*A*	*D*—H···*A*
N2—H2···O1*B*^i^	0.88	2.15	2.959 (14)	152
N2—H2···O1*A*^i^	0.88	2.27	3.089 (16)	155
N5—H5···O3*B*	0.88	2.22	3.075 (10)	163
N5—H5···O2*A*	0.88	2.40	3.271 (11)	172
N8—H8···O3*A*^i^	0.88	2.21	3.073 (12)	168
N11—H11···O2*B*	0.88	2.26	3.129 (10)	168
N11—H11···O2*A*	0.88	2.27	3.093 (11)	156

Symmetry code: (i) *x*, −*y*+1/2, *z*−1/2.
